# Supplementary material for: A Stress Syndrome Prototype Reflects Type 3 Diabetes and Ischemic Stroke Risk: The SABPA Study
Source: Biology (Basel). 2021 Feb 18;10(2):162. doi: 10.3390/biology10020162 (PMC7922484; doi:10.3390/biology10020162)

biology

Supplementary Methodology

Type of the Paper: Article

Title: A Stress syndrome prototype reflects type 3 diabetes and ischemic stroke risk: The SABPA study

[1]Leoné Malan, [2]Mark Hamer, [1,3]Roland von Känel, [4]Roelof D van Wyk, [5]Anne E Sumner, [6]Peter M Nilsson, [7,8]Gavin W Lambert, [9]Hendrik S Steyn, [10]Casper J Badenhorst, [1]Nico T Malan

Affiliations:

1 Hypertension in Africa Research Team (HART), North-West University, Potchefstroom 2520, South Africa; Roland.VonKaenel@usz.ch (R.v.K.); nico.t.malan@gmail.com (N.T.M.)
2 Division of Surgery & Interventional Science, Faculty of Medical Sciences, University College London, WC1E 6BT London UK; m.hamer@ucl.ac.uk
3 Department of Consultation-Liaison Psychiatry and Psychosomatic Medicine, University Hospital Zurich, 8091 Zurich, Switzerland
4 Surgical Ophthalmologist, 85 Peter Mokaba Street, Potchefstroom 2531, South Africa; rdvisu@gmail.com
5 Section on Ethnicity and Health, Diabetes, Endocrinology and Obesity Branch, National Institute of Diabetes and Digestive and Kidney Diseases, and National Institute of Minority Health and Health Disparities, National Institutes of Health, Bethesda, MD 20892-1612, USA; anne.sumner@nih.gov
6 Department of Clinical Sciences, Lund University, Malmö, SE-205 02 Sweden; peter.nilsson@med.lu.se
7 Iverson Health Innovation Research Institute, Swinburne University of Technology, Hawthorn, *VIC* 3122 Australia; glambert@swin.edu.au
8 Baker Heart & Diabetes Institute, Melbourne 3004, Australia
9 Statistical Consultation Services, North-West University, Potchefstroom 2520, South Africa; faans.steyn@gmail.com
10 Anglo American Corporate Services, Sustainable Development Department, Johannesburg 2017, South Africa; dr.cas.badenhorst@gmail.com
* Correspondence: leonemalan6@gmail.com; leone.malan@nwu.ac.za
Tel.: +27-(0)-733765321

METHODOLOGY

1. Perception of Stroop test stressfulness
2. Ambulatory blood pressure
3. Time-domain heart-rate-variability (HRV)
4. Carotid bifurcation stenosis
5. Telomere length
6. Mitochondrial DNA – MutPred Load
7. HIV status
8. Table S1
9. Figure S1

METHODOLOGY

1. *Perception of Stroop test stressfulness*

Perception of test stressfulness was assessed on a 7-point Likert scale. After completion of each task, participants reported their overall distress according to the question "How stressful was the task you just completed?" on a 7-point Likert scale ranging from not severe to highly severe.

2. *Ambulatory blood pressure*

The blood pressure cuff was fitted to the non-dominant arms using an appropriate cuff size. Participants were asked to record occurrences of stress and any disturbances on their 24h ambulatory diary card. In the SABPA study, blood pressure measures were obtained every 30 minutes during the day (08:00-22:00) and hourly during the night (22:00-06:00) to measure blood pressure without too much disturbance/inconvenience during the normal school working day and after school extracurricular activities [26]. Participants were asked to record occurrences of stress and any disturbances on their 24h ambulatory diary card. The 24h successful inflation rate was 77.1% (±12.8) in Stressed individuals and 83.1% (±10.2) in no-Stressed individuals. The data were analyzed with the CardioVisions 1.19 Personal Edition software (Meditech, Budapest, Hungary). Hypertensive status according to the 2018 European Society of Cardiology Guidelines was classified as 24h SBP ≥ 130 mm Hg and/or DBP ≥ 80 mm Hg.

3. *Time-domain heart-rate-variability (HRV)*

Time-domain HRV analysis was computed to assess spontaneous oscillations resulting from sinus node depolarization. Two-channel ECG recordings over 24h were used to assess time-domain HRV (Cardiotens CE120®, Meditech, Budapest Hungary) were obtained according to a preset program for 20s at 5-min intervals. The software program automatically filtered out ventricular, supraventricular as well as artefacts in RR intervals, and HRV outliers had been manually removed. Time-domain analysis marker included the standard deviation of the normal-to-normal (NN) intervals between adjacent QRS complexes, which equal the square root of variance (SDNN ms). SDNN is regarded as the best overall prognostic tool for values <50 ms are indicative of highly depressed HRV, those between 50-100 ms indicate moderately depressed HRV and those >100 ms are classified as normal. SDNN further reflects vagus nerve-mediated autonomic control of the heart.

4. *Carotid bifurcation stenosis*

A trained cardiovascular scientist determined carotid intima media thickness and bifurcation stenosis at 3-year follow-up. Re-evaluation and calculation was done by an independent Echocardiogram Technologist and added as covariate in retinal vessel regression analyses models. Intra-observer variability was 0.04 mm between two measurements made 4 weeks apart on 10 participants. High resolution ultrasound images were acquired from at least two optimum angles of the common carotid artery segments (Sonosite Micromaxx ultrasound system (SonoSite Inc., Bothell, WA, USA) and 6-13 MHz linear array transducer] using the Rudy Meijer protocol. The images were digitized and imported into the Artery Measurement Systems automated software (AMS) II v1.139 (Gothenburg, Sweden) for dedicated analysis of the carotid artery as ischemic stroke risk marker [15].

5. *Telomere length* [21]

Before telomere analyses all samples were diluted to the concentration of 10 ng/µl. Reference DNA samples were prepared, and all isolated DNAs were mixed together in equal proportions representing the average of all analyzed patients (N=255). LTL was assessed by multiplex Quantitative Real-Time PCR (Q-PCR) (Fani et al., 2020). Five concentrations of a reference DNA sample spanning a 75-fold range of DNA concentrations were prepared by serial dilution and analyzed in triplicate; these reactions provided the data for the generation of the standard curves used for relative quantification. All experimental DNA samples were assayed in triplicate. All PCRs were performed with CFX96 Touch™ Real-Time PCR Detection System (Bio-Rad, Hercules, CA, USA) in a 25 µl volume. PCR reactions comprised of 20 µl master mix (including 5 µl of 5x HOT FIREPol® EvaGreen® qPCR Mix Plus (no ROX) (Solis BioDyne, Tartu, Estonia) and 5 µl of each experimental DNA sample.

For multiplex Q-PCR, the telomere primer pair telg:
ACACTAAGGTTTGGGTTTGGGTTTGGGTTTGGGTTAGTGT and telc:
TGTTAGGTATCCCTATCCCTATCCCTATCCCTATCCCTAACA (final concentrations
900 nM each), were combined with primers for the single copy gene human beta-globin (hbg); hbgu: CGGCGGCGGGCGGCGCGGGCTGGGCGGCTTCATCCACGTTCACCTTG and hbgd: GCCCGGCCCGCCGCGCCCGTCCCGCCGGAGGAGAAGTCTGCCGTT (final concentrations 500 nM each), to give PCR products of 79 bp and 106 bp, respectively. All primers were manufactured by Integrated DNA Technologies (Coralville, IA, USA). The thermal cycling profile was Stage 1: 15 min at 95°C; Stage 2: 2 cycles of 15s at 94°C, 15 s at 49°C; and Stage 3: 32 cycles of 15 s at 94°C, 10 s at 62°C, 15 s at 73°C with signal acquisition, 10 s at 84°C, 15 s at 87°C with signal acquisition. The 73°C reads provided the Ct values for the amplification of the telomere template (in early cycles where the hbg signal is still at baseline); the 87°C reads the Ct values for the amplification of the hbg template (at this temperature there is no signal from the telomere PCR product, because it is fully melted). After thermal cycling and raw data collection were complete, C_T values generated by CFX Manager™ Software Version 1.6 (Bio-Rad, Hercules, CA, USA) were exported to REST© software (Relative Expression Software Tool), version REST-384. PCR efficiency for each primer set was calculated by serial dilutions of reference DNA using the REST software tool. The relative LTL per cell was also calculated using the REST software as the ratio (T/S) between relative content of telomere PCR product (T) and human beta-globin PCR product (S). The LTL was calculated for each individual participant relative to the average LTL of the total analyzed group (344 participants).

6. *Mitochondrial DNA (mtDNA) – MutPred Load*

DNA for sequencing was extracted from whole blood samples. Sequencing of the entire mtDNA molecule was done using the Ion Torrent PGM, as described elsewhere [42]. Whole mtDNA sequences have been deposited in the sequence read archive (SRA) of NCBI under BioProject accession number PRJNA403942 (https://www.ncbi.nlm.nih.gov/ bioproject/403942). Consensus variants were called against the revised Cambridge reference sequence (rCRS). MtDNA haplogroups were assigned according to the mtDNA variant list of each participant, using Haplogrep 2.0.y We summed the MutPred scores generated for each of the non-synonymous variants after calculating "MutPred mutational loads" on an individual's mtDNA; and adjusted these for the position of the sequence in the phylogeny, ultimately calculating "MutPred adjusted loads". Many different tools for predicting pathogenicity of mtDNA variants exist, but MutPred and SNPs & GO outperformed all other methods, which included PolyPhen2, SIFT and SNAP.T

7. *Human immunodeficiency virus (HIV).*

HIV positive status was determined (*Supplementary methodology*) with a rapid antibody test in plasma (First response kit. PMC Medical, Daman, India) and confirmed with the Pareekshak test

(BHAT Bio-Tech, Bangalore, India). Pre- and post-counselling for HIV positive status was done by a trained registered nurse.

8. *Table S1: Pearson correlations between cognitive executive control and dementia risk markers*

Table S1: Pearson correlations between cognitive executive control [29] and dementia risk markers in Stressed and no-Stress individuals.

	Stressed group (N=**159)**		non-Stressed group (N=**105)**	
S100B (ng/ml)	**r = -0.22**	**p = 0.009**	r = 0.02	p = 0.820
Waist circumference (cm)	**r = 0.22**	**p = 0.007**	r = -0.16	p = 0.124
Von Willebrand factor (%)	**r = -0.27**	**p = 0.006**	r = -0.16	p = 0.117
24h HRV-SDNN (ms)	r =- 0.08	p = 0.347	r = -0.07	p = 0.473
HOMA-IR	**r = 0.19**	**p = 0.023**	r = -0.15	p = 0.135
Telomere length (ng/µl)	**r = 0.25**	**p = 0.002**	**r = 0.26**	**p = 0.011**
Neuron-specific enolase (ng/ml)	r = 0.05	p = 0.517	r = -0.07	p = 0.481
C-reactive protein (mg/l)	r = -0.14	p = 0.083	r = -0.03	p = 0.793
Perception of Stroop test stressfulness	**r = -0.19**	**p = 0.020**	r = -0.02	p = 0.808

The values are displayed as the correlation coefficient and the p-value. Dementia risk markers included dementia-related risk markers indicating cardiometabolic perturbations, vascular dysregulation and neurodegeneration, were specified and included in the current investigation: poorer cognitive executive functioning control [30]; neuronal glia injury (increased S100B, NSE) [19]; increased central obesity or waist circumference (WC) [32]; endothelial dysfuntion (von Willebrand factor (VWF)) [33]; sympathovagal imbalance or depressed time-domain heart-rate-variability (HRV) [34]; increased insulin resistance/HOMA-IR [8]; -inflammation (c-reactive protein/CRP) [35] and shorter telomeres [21]. HRV-SDNN, time-domain 24h heart-rate-variability standard deviation of the normal-to-normal (NN) intervals between adjacent QRS complexes which equal the square root of variance.

9. Figure S1: Presenting the apparatus and method to determine retinal vessel imaging (Figure S1a,b); and the Stroop test (Figure S1c,d), which was used as measure of cognitive executive functioning control.

See page 5:

S1a

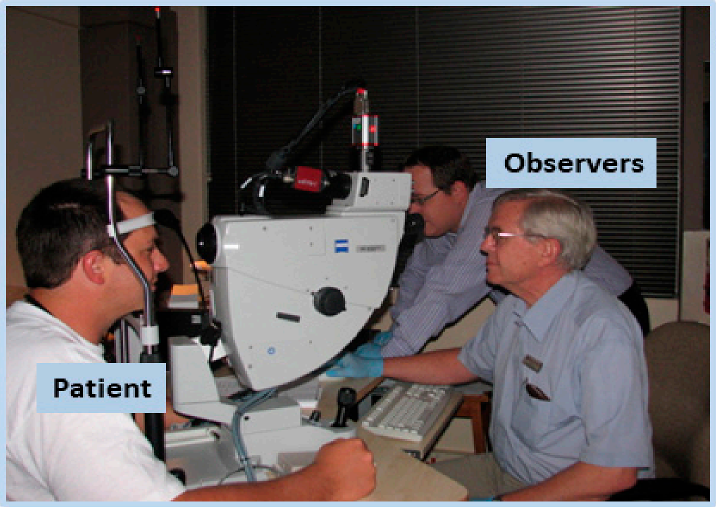

S1b

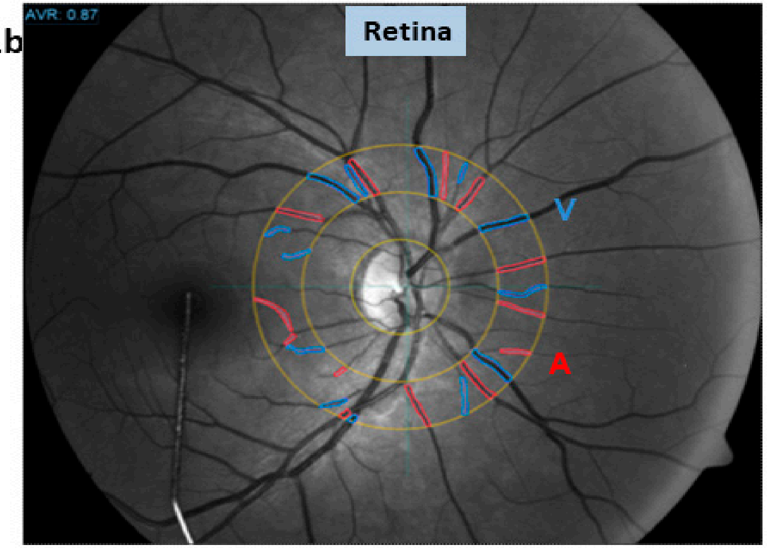

S1c

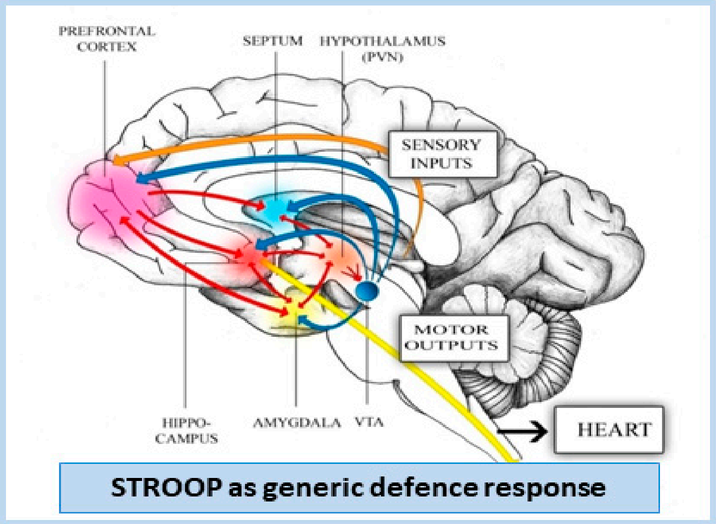

S1d

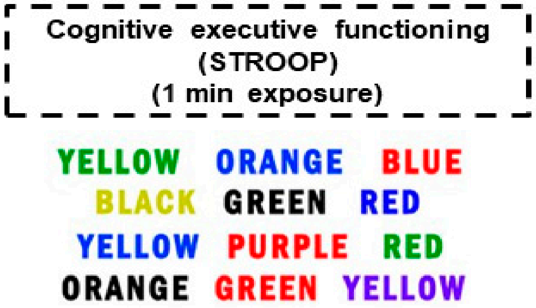

Supplement: Supplementary file 1 [file biology-10-00162-s001.pdf]
